# Empagliflozin and dapagliflozin decreased atrial monoamine oxidase expression and alleviated oxidative stress in overweight non-diabetic cardiac patients

**DOI:** 10.1007/s11010-024-05076-z

**Published:** 2024-07-23

**Authors:** Loredana N. Ionică, Darius G. Buriman, Adina V. Lința, Raluca Șoșdean, Ana Lascu, Caius G. Streian, Horea B. Feier, Lucian Petrescu, Ioana M. Mozoș, Adrian Sturza, Danina M. Muntean

**Affiliations:** 1 Doctoral School Medicine-Pharmacy, “Victor Babeș” University of Medicine and Pharmacy From Timișoara, Eftimie Murgu Sq. No. 2, 300041 Timișoara, Romania; 2 Department V Internal Medicine – 1st Clinic of Medical Semiotics, “Victor Babeș” University of Medicine and Pharmacy From Timișoara, Eftimie Murgu Sq. No. 2, 300041 Timișoara, Romania; 3 Centre for Translational Research and Systems Medicine, “Victor Babeș” University of Medicine and Pharmacy From Timișoara, Eftimie Murgu Sq. No. 2, 300041 Timișoara, Romania; 4 Department III Functional Sciences – Chair of Pathophysiology, “Victor Babeș” University of Medicine and Pharmacy From Timișoara, Eftimie Murgu Sq. No. 2, 300041 Timișoara, Romania; 5 Department VI Cardiology, Clinic of Cardiology, “Victor Babeș” University of Medicine and Pharmacy From Timișoara, Eftimie Murgu Sq. No. 2, 300041 Timișoara, Romania; 6 Institute of Cardiovascular Diseases Timișoara, “Victor Babeș” University of Medicine and Pharmacy From Timișoara, Eftimie Murgu Sq. No. 2, 300041 Timișoara, Romania; 7 Department VI Cardiology – Clinic of Cardiovascular Surgery, “Victor Babeș” University of Medicine and Pharmacy From Timișoara, Eftimie Murgu Sq. No. 2, 300041 Timișoara, Romania

**Keywords:** Empagliflozin, Dapagliflozin, Monoamine oxidase, Atrial oxidative stress, Overweight, Non-diabetic patients, Echocardiography

## Abstract

The sodium-glucose-cotransporter 2 inhibitors (SGLT2i) are the blockbuster antidiabetic drugs that exert cardiovascular protection via pleiotropic effects. We have previously demonstrated that empagliflozin decreased monoamine oxidase (MAO) expression and oxidative stress in human mammary arteries. The present study performed in overweight, non-diabetic cardiac patients was aimed to assess whether the two widely prescribed SGLT2i decrease atrial MAO expression and alleviate oxidative stress elicited by exposure to angiotensin 2 (ANG2) and high glucose (GLUC). Right atrial appendages isolated during cardiac surgery were incubated ex vivo with either empagliflozin or dapagliflozin (1, 10 µm, 12 h) in the presence or absence of ANG2 (100 nm) and GLUC (400 mg/dL) and used for the evaluation of MAO-A and MAO-B expression and ROS production. Stimulation with ANG2 and GLUC increased atrial expression of both MAOs and oxidative stress; the effects were significantly decreased by the SGLT2i. Atrial oxidative stress positively correlated with the echocardiographic size of heart chambers and negatively with the left ventricular ejection fraction. In overweight patients, MAO contributes to cardiac oxidative stress in basal conditions and those that mimicked the renin–angiotensin system activation and hyperglycemia and can be targeted with empagliflozin and dapagliflozin, as novel off-target class effect of the SGLT2i.

## Introduction

Inhibitors of the sodium-glucose cotransporter 2 (SGLT2i) have become an essential part of the type 2 diabetes mellitus therapeutic arsenal, with improved cardiovascular outcomes in all patients [1]. Indications for their use have expanded beyond glucose reduction to include the cardiac protection in patients with heart failure (HF) with reduced and preserved ejection fraction, regardless the presence of diabetes, as demonstrated by a series of landmark large clinical trials (recently reviewed in Refs. [2, 3]). Accordingly, empagliflozin and dapagliflozin are currently part of the foundational HF therapeutics, mainly due to their significant benefits in reducing hospitalizations but also due to diminishing cardiovascular mortality and increasing life quality in patients with chronic HF [4–6]. Extensive experimental and clinical evidence have unequivocally documented the multifaceted benefits of these drugs that extend far beyond the glucose control [7, 8] and are supported by several “off-target” direct cardiac effects, such as the antioxidant, anti-inflammatory, metabolic, anti-fibrotic, ones, besides the hemodynamic changes related to natriuria and glucosuria [9–11].

Oxidative stress has been acknowledged as a central pathophysiological mechanism that disrupts the cardiomyocyte homeostasis in the vast majority of cardiovascular diseases [12]. Increased generation of reactive oxygen species (ROS), particularly in the aged heart [13], plays an important role in the genesis of subcellular defects, e.g., mitochondrial dysfunction and disruption of calcium signaling, which contribute to the impairment of systolic and diastolic function [14]. Furthermore, it has been unequivocally demonstrated that oxidative stress interacts in a complex manner with the low-grade inflammation state to promote a vicious circle responsible for both cardiac remodeling and HF progression [15].

Obesity is a complex, highly heritable multifactorial disease defined as excessive fat accumulation that increases the body mass index (BMI > 30 kg/m^2^) and is associated with both heightened oxidative stress and subclinical inflammation [16]. Not only obesity may impair cardiovascular health but also overweight (BMI = 25–30 kg/m^2^) is a state of altered redox homeostasis, albeit a cause–effect relationship is not easy to establish in either condition. In any case, the increased visceral abdominal fat is the major culprit linked to cardiovascular risk and morbidity via several direct and indirect pathomechanisms [17].

Mitochondrial dysfunction and the redox imbalance have been systematically implicated in the development of heart failure [18] and diabetic cardiomyopathy [19] with mitochondria being both major sources and targets of the reactive oxygen species (ROS) as well as the main site of the ROS-induced ROS release phenomenon [20]. However, the individual contribution of the mitochondrial ROS sources to the cardiac oxidative stress is far from being elucidated.

A relevant source of constant hydrogen peroxide (H_2_O_2_) production in the cardiovascular system is monoamine oxidase (MAO) with 2 isoforms, MAO-A and MAO-B located at the outer mitochondrial membrane, which catalyze the oxidative deamination of biogenic monoamines and neurotransmitters (for an excellent comprehensive review see Ref. [21]). We have demonstrated that MAO is present in the atrial tissue of patients with coronary artery disease with and without diabetes with the predominance of the MAO-B isoform, particularly in the former group [22]. We have also recently reported the contribution of MAO-related vascular oxidative stress to the endothelial dysfunction in human mammary arteries harvested from overweight patients with HF with mildly reduced ejection fraction undergoing revascularization procedures; in this pilot study, ex vivo incubation with empagliflozin alleviated endothelial dysfunction, mitigated oxidative stress, and decreased vascular expression of both MAO-A and MAO-B [23].

The present study performed in atrial tissue harvested from overweight, non-diabetic patients with HF and indication for elective cardiac surgery was aimed to assess whether empagliflozin and dapagliflozin can mitigate MAO isoforms expression and the oxidative stress elicited by acutely ex vivo incubation with angiotensin 2 (ANG2) and high glucose (GLUC), respectively. Furthermore, whether oxidative stress correlates with the echocardiographic parameters was also investigated.

## Materials and methods

The study was conducted in accordance with the principles of the Declaration of Helsinki and the protocol was approved by the Committee for Research Ethics of “Victor Babeș” University of Medicine and Pharmacy of Timișoara, Romania (No. 05/10.02.2023). Written informed consent was obtained from all patients prior to surgery.

Atrial samples were harvested from patients undergoing open-heart surgery once cardiopulmonary bypass was established by resecting the tip of the right atrial appendage; the atrial samples were further transferred to the laboratories of the Centre for Translational Research and Systems Medicine for all the ex vivo measurements.

The present pilot study included 24 consecutive non-diabetic patients (20 males and 4 females) who were overweight (BMI = 28.98 ± 0.31 kg/m^2^) and diagnosed with all types of HF (ejection fraction, EF = 43.47 ± 11), i.e., HF with preserved (HFpEF), mildly reduced (HFmrEF), and reduced (HFrEF). The main diagnostics (indications for elective cardiac surgery) were aortic and mitral valvular diseases, in association with coronary artery disease, hypertension, and various arrhythmias (atrial fibrillation, atrial flutter, premature ventricular contractions). All patients were treated with ACE inhibitors or ARBs, β—blockers, calcium channel blockers, diuretics, and statins. Antiplatelet and anticoagulant therapy were stopped 5 days prior to the intervention.

The characteristics of the enrolled patients are listed in Table 1.

**Table 1:** Characteristics of the study group

Parameter	Value
Age (years)	62.20 ± 12.54
Sex (M/F)	20/4
BMI (kg/m^2^)	28.98 ± 0.31
Systolic blood pressure (mmHg)	132.91 ± 6.5
Diastolic blood pressure (mmHg)	75.20 ± 7.58
Heart rate (b/min)	75.25 ± 7.61
Erythrocyte sedimentation rate (mm/h)	23.68 ± 4.13
Red blood count (mil/mm^3^)	4.56 ± 0.64
Hemoglobin (g/dL)	13.54 ± 1.67
White blood count (*10^3^/mm^3^)	7.80 ± 2.27
Platelets (*10^3^/mm^3^)	220.75 ± 68.89
Creatinine (mg/dL)	1.07 ± 2.7
FPG (mg/dL)	117.9 ± 3.1
ALAT (U/L)	37.45 ± 1.9
ASAT (U/L)	38.08 ± 3.83
Na^+^ (mmol/L)	138,75 ± 2.41
K^+^ (mmol/L)	3.89 ± 0.46
LVEF (%)	43.47 ± 11
LVEDV (ml)	138.87 ± 38.56
LAD (cm)	4.61 ± 0.48
RVD (cm)	2.98 ± 0.61
IVS (cm)	1.49 ± 0.29
LVEDD (cm)	5.14 ± 0.77

Data are expressed as mean ± S.E.M. *BMI* Body Mass Index, *PCV* Packed Cell Volume, *FPG* Fasting Plasma Glucose, *ALAT* Alanine Aminotransferase, *ASAT* Aspartate Aminotransferase, *LVEF* Left Ventricular Ejection Fraction, *LVEDV* Left Ventricular End Diastolic Volume, *LAD* Left Atrium Diameter, *RVD* Right Ventricular Diameter, *IVS* Interventricular Septum, *LVEDD* Left Ventricular End Diastolic Diameter

### Assessment of oxidative stress in organ culture

In order to mimic the activation of the renin–angiotensin–aldosterone system and uncontrolled diabetes mellitus, atrial tissue samples were cleaned and incubated for 12 h at 37 °C in a culture medium containing 0.1% bovine serum albumin in the presence or absence of ANG2 (100 nm) and high GLUC (400 mg/dL), with or without empagliflozin (1 and 10 µm) or dapagliflozin (1 and 10 µm). The tissue was then embedded in Tissue Tek for the assessment of superoxide ion using the dihydroethidium (DHE) probe in confocal microscopy (Olympus Fluoview FV1000, laser excitation at 488 nm and emission at 610 nm) as described in Ref. [24]. Hydrogen peroxide production was assessed in spectrophotometry with the Ferrous Oxidation-Xylenol Orange (FOX) assay (PeroxiDetect Kit, Sigma Aldrich-Merck) according to a method described in Ref. [25].

### Assessment of monoamine oxidases expression

Both MAOs’ gene and protein expressions were assessed as previously described in Ref. [24].

#### MAO gene expression assessment

In order to assess the MAOs gene expression, the RT-PCR technique was performed in samples dissected from the right atrial appendages. Total RNA was isolated (Aurum Total RNA Mini Kit, Biorad) and used for reverse transcription (iScript Advanced cDNA Synthesis Kit, Biorad) and primers against MAO-A and MAO-B were designed using the sequence information from the NCBI database (5’ → 3’): human MAO-A fw: CTG ATC GAC TTG CTA AGC TAC, human MAO-A rev: ATG CAC TGG ATG TAA AGC TTC. The housekeeping gene (EEF2, eukaryotic elongation factor 2) and its primers were as follows (5’ → 3’): EEF2 fw: GAC ATC ACC AAG GGT GTG CAG and EEF2 rv: GCG GTC AGC ACA CTG GCA TA.

#### MAO protein expression assessment

Tissue expression of MAOs isoforms was quantified in frozen atrial sections using the MAO-A (Abcam, ab126751) and MAO-B (Abcam, ab175136) primary antibodies and Alexa Fluor-labeled secondary goat anti-rabbit antibody (Invitrogen, A32731), while DAPI (Santa Cruz, SC3598) was used for the nuclear staining. The slides were examined in confocal microscopy (Olympus Fluoview FV1000) and image analysis was performed using the Image J software.

### Statistics

Statistical data processing was performed with the GraphPad Prism software Version 9.3.1 (GraphPad, USA). Data are presented as mean ± SEM and were analyzed using one-way ANOVA. Pearson’s linear correlation was used to determine correlations between oxidative stress and the echocardiographic parameters. Values of *p* < 0.05 were considered statistically significant.

## Results

### Empagliflozin and dapagliflozin alleviated oxidative stress in human atrial tissue

**Fig. 1:**
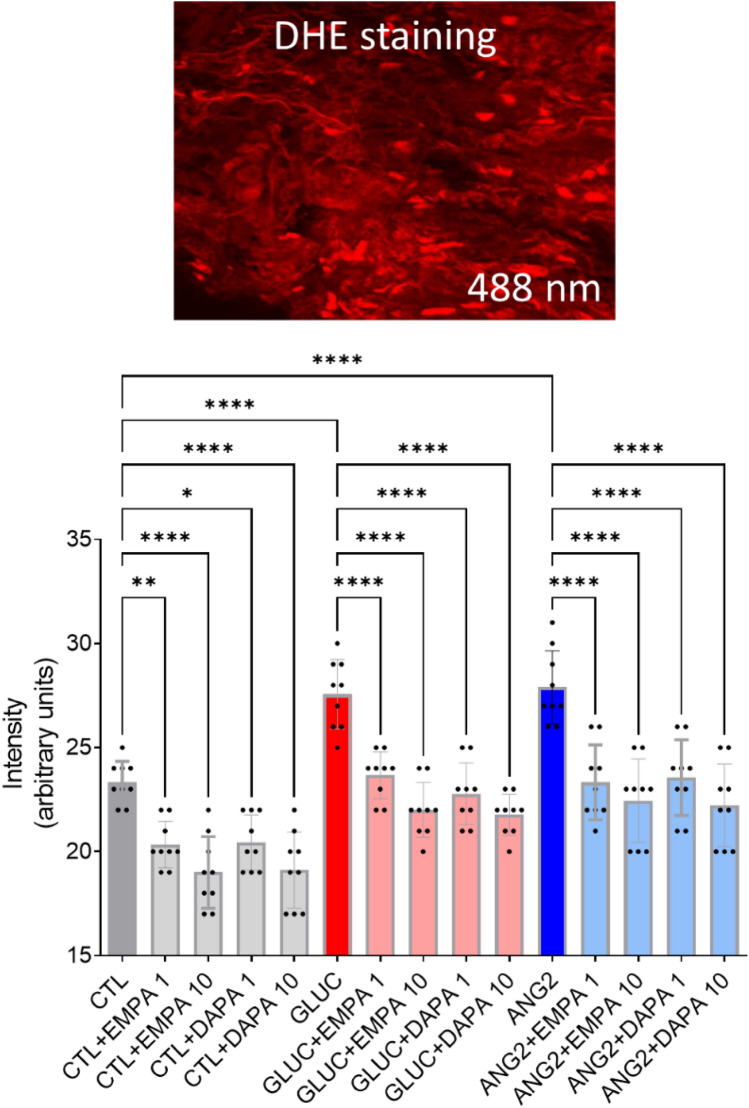
Empagliflozin and dapagliflozin mitigated oxidative stress level in human atrial tissue—DHE staining after incubation with ANG2 and GLUC (*n* = 24). *p < 0.05, **p < 0.01, ***p < 0.001, ****p < 0.0001

**Fig. 2:**
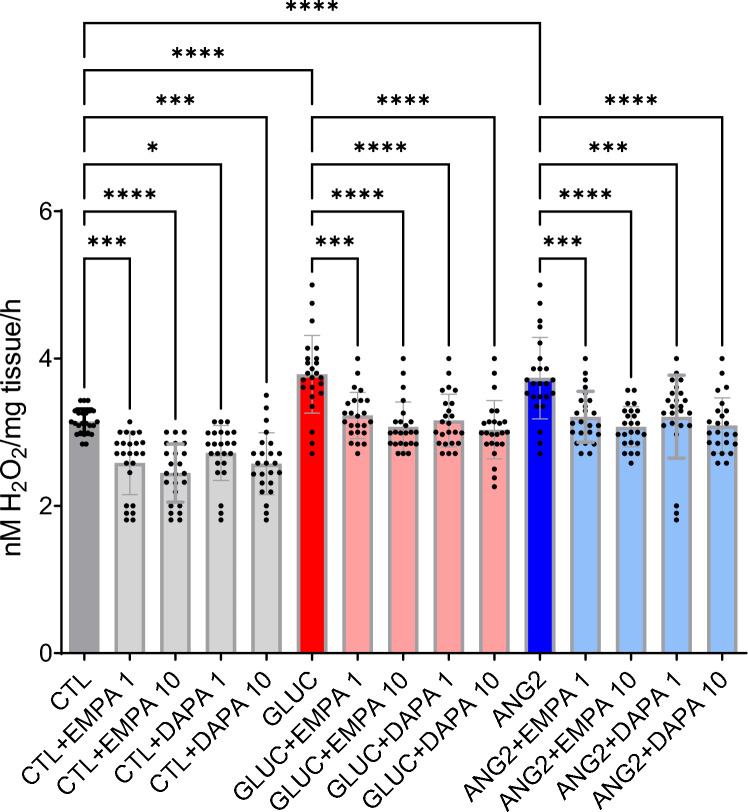
Empagliflozin and dapagliflozin decreased oxidative stress level in human atrial tissue—FOX assay after incubation with ANG2 and GLUC (*n* = 24). *p < 0.05, **p < 0.01, ***p < 0.001, ****p < 0.0001

We examined the acute effect of empagliflozin (EMPA) and dapagliflozin (DAPA) on ROS generation in human atrial tissue after ex vivo incubation with ANG2 (100 nm) and GLUC (400 mg/dL) and assessed the ROS levels by two methods. As revealed by DHE staining (Fig. 1) and FOX assay (Fig. 2), oxidative stress was augmented by ANG2 and GLUC. Co-incubation with the SGLT2i (both concentrations) was able to significantly attenuate this effect.

Importantly, the effects of both drugs were also present in the control samples (not stimulated with GLUC or AII), demonstrating the occurence of oxidative stress in overweight patients with all spectrum of heart failure (reduced, mildly reduced, and preserved ejection fraction), thus, strongly supporting the rationale for the early administration of SGLT2i in overweight patients, view the local beneficial effects.

### SGLT2i do not exert ROS-scavenger properties

**Fig. 3:**
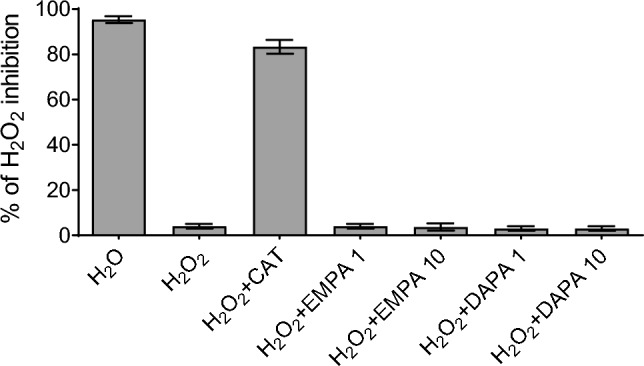
Empagliflozin and dapagliflozin (1, 10 µm) do not exhibit ROS-scavenger properties at FOX assay (the antioxidant activity of empagliflozin and dapagliflozin was compared with the one of catalase—100 U/mL) in the presence of H_2_O_2_—100 mm)

We also tested whether EMPA and DAPA act as a ROS scavengers and showed that they have very little antioxidant capacity as compared to catalase, a classical ROS scavenger (Fig. 3). This observation suggests that SGLT2i are not able to directly reduce oxidative stress but through the modulation of intracellular ROS sources.

### SGLT2i decreased MAO expression in human atrial tissue

**Fig. 4:**
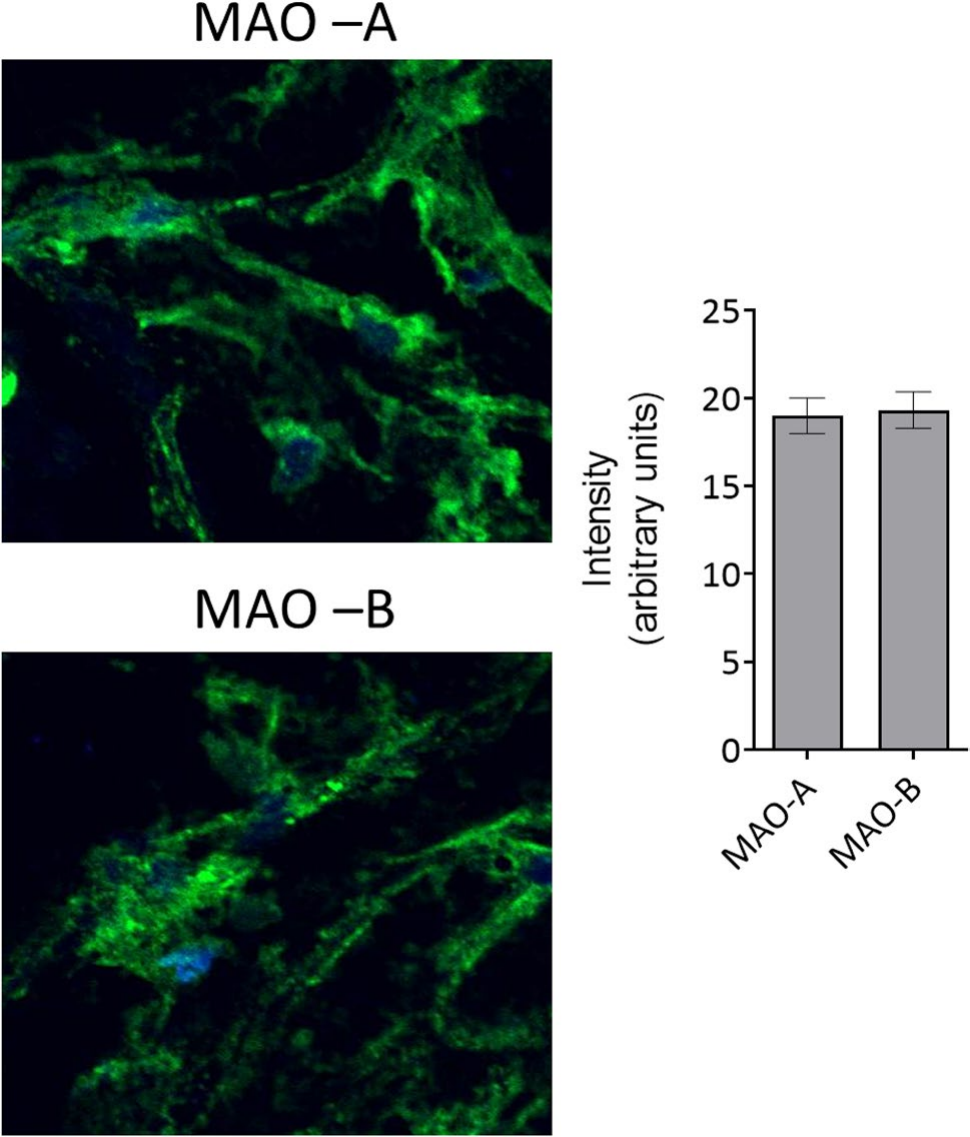
MAO-A and MAO-B protein expression in human atrial samples (20 × , blue = DAPI, green = MAO-A and B)

Since acute ex vivo incubation with EMPA and DAPA was able to reduce atrial ROS generation, we further investigated whether SGLT2i modulates MAO expression in these samples. We observed that both MAO isoforms are equally expressed in the human atrial tissue harvested from the overweight patients (Fig. 4).

**Fig. 5:**
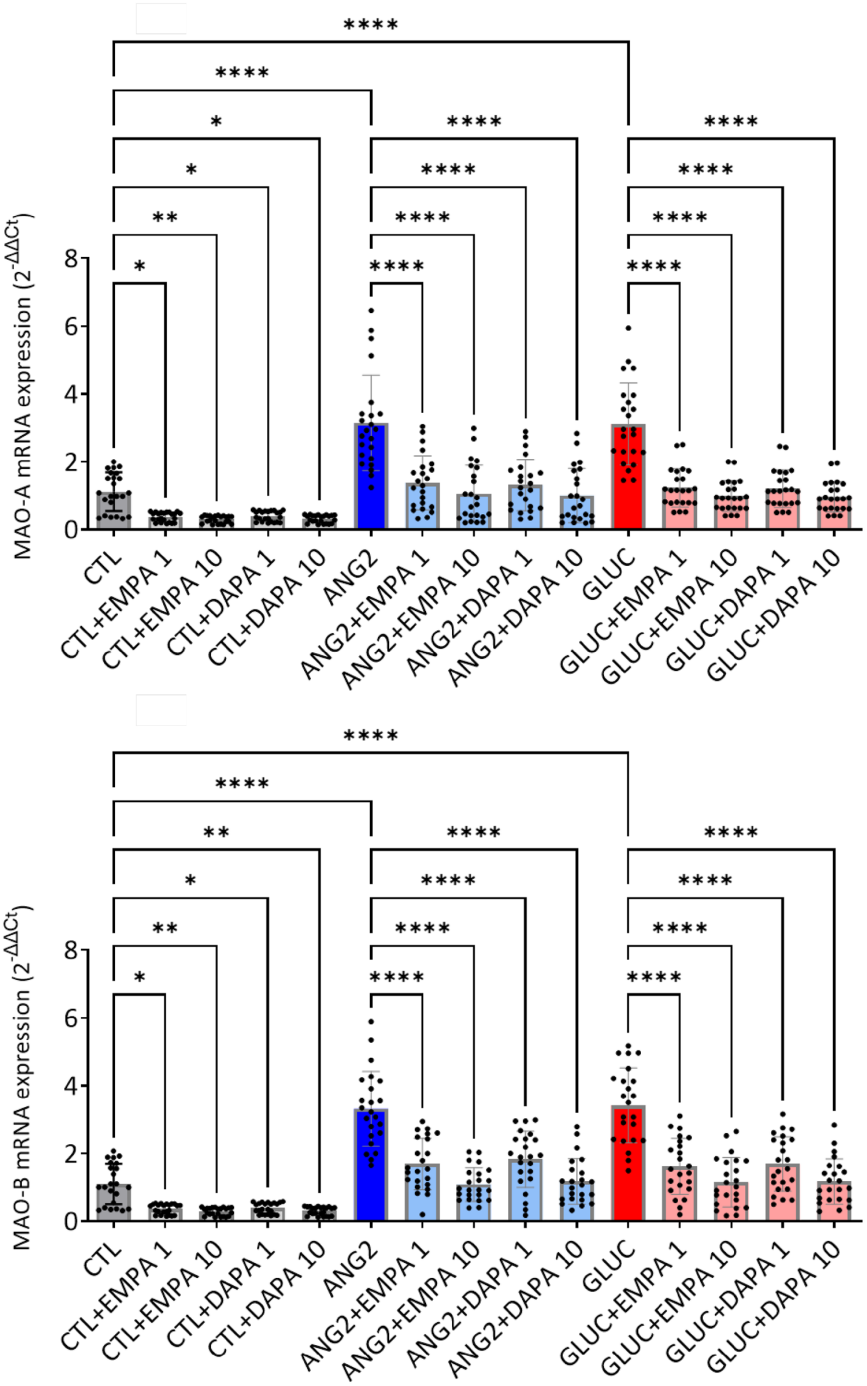
Empagliflozin and dapagliflozin reduced MAO-A and MAO-B mRNA gene expression after incubation with ANG2 and GLUC (*n* = 24). *p < 0.05, **p < 0.01, ***p < 0.001, ****p < 0.0001

In vitro exposure to EMPA and DAPA reduced MAO-A and MAO-B gene expression in atrial samples stimulated ANG2 and GLUC (Fig. 5). As for the oxidative stress, the effect was also observed in the control, non-stimulated samples.

### Oxidative stress significantly correlated with the main echocardiographic parameters

**Fig. 6:**
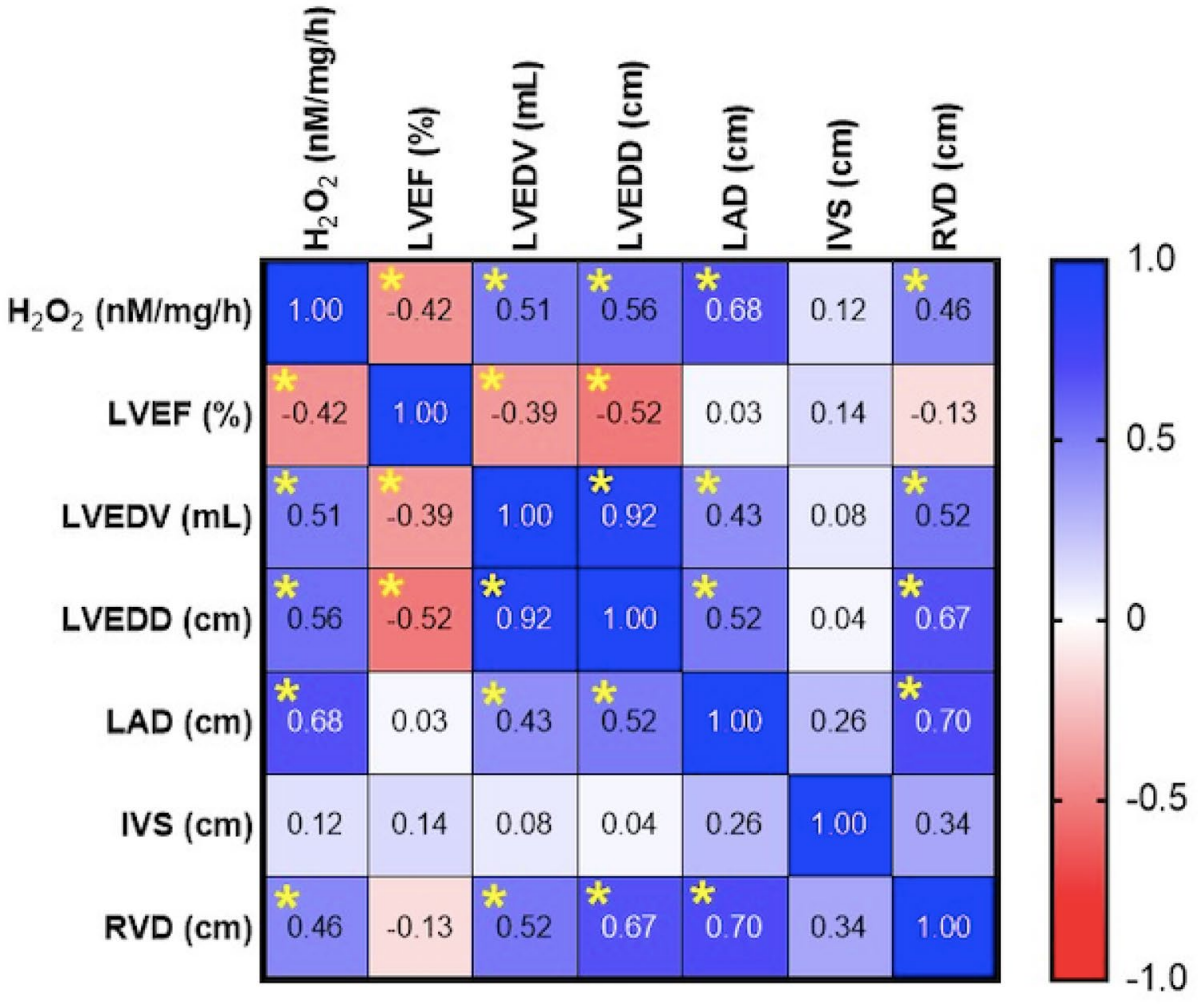
Pearson *r* matrix correlation between ROS atrial level and echocardiographic parameters (LV ejection fraction—LVEF, LV end-diastolic volume—LVEDV, LA diameter—LAD, RV diameter—RVD, interventricular septum—IVS, LV end-diastolic-diameter- LVEDD), *n* = 24, *p < 0.05

Correlation analysis was performed to evaluate a potential relation between the echocardiographic parameters (LV ejection fraction—LVEF, LV end-diastolic volume—LVEDV, LA diameter—LAD, RV diameter—RVD, interventricular septum—IVS, LV end-diastolic-diameter—LVEDD) and the degree of oxidative stress (H_2_O_2_ level measured by FOX assay) in the analyzed samples. We observed a positive correlation between ROS levels and the LA diameter (0.68), LVED diameter (0.56), and LVED volume (0.51) and interestingly, also with the RV diameter (0.46). At variance, a significant negative correlation was noticed between ROS and the LVEF (−0.42, Fig. 6).

## Discussion

The role of impaired redox signaling and of oxidative stress-mediated mitochondrial dysfunction in the pathogenesis of heart failure and metabolic pathologies (obesity, metabolic syndrome, diabetes) has been systematically reported in the literature. In the recent years, the effects of the novel classes of antidiabetics have been systematically investigated in order to gain knowledge about the mechanisms underlying their protective effects in the cardiovascular system (reviewed in Refs. [26–28]).

The SGLT2 inhibitors are disease-modifying drugs that are currently transforming the therapeutic landscape of heart failure via pleiotropic “off-target” cardiac effects, beyond the glycemia control [2]. Among these, their antioxidant effect has been unequivocally demonstrated along the time both in the setting of diabetes [29] and heart failure [30]; however, the sources of cardiac ROS targeted by SGLT2i are far from being fully elucidated.

In the present study, we investigated the effects of empagliflozin and dapagliflozin, the two SGLT2 inhibitors included in the HF guidelines, on oxidative stress and MAO expression in human atrial samples harvested from overweight non-diabetic patients with HF with all spectrums of ejection fraction. The major findings are the reduction of ROS generation and mitigation of MAO gene expression in right atrial appendages after acute ex vivo exposure to ANG2 and high GLUC, conditions that mimic the RAAS activation (a central pathomechanism in HF) and the uncontrolled diabetes, respectively. Importantly, these effects were demonstrated for both EMPA and DAPA, as a class effect of SGLT2i, and also, at a concentration relevant to the one present in human plasma (1 microM) after oral administration. Interestingly, we have noticed that the SGLT2i alleviated oxidative stress both in control (non-stimulated) and stimulated (ANG2, high glucose) atrial samples, an effect that was more significant for EMPA as compared to DAPA at the above-mentioned concentration. Similarly, mitigation of the MAO gene expression was also observed in control samples, the magnitude being equivalent for EMPA and DAPA at 1 microM. The observation of a beneficial effect on ROS production and MAO expression was previously reported for EMPA (albeit only in the high concentration of 10 microM) when acutely applied ex vivo on mammary arteries rings harvested from overweight patients [23], thus demonstrating that MAO-related cardiovascular oxidative stress is already present in overweight patients (prior to the occurence of obesity). Of interest, it has been reported in an experimental model of prediabetes in young rats that mitochondrial oxidative stress in cardiomyocytes contributes to the depression of soluble guanylyl cyclase/PKG activity and impairment of relaxation with subsequent diastolic dysfunction, which preceded the coronary endothelial dysfunction (indicated by preservation of the dilator response to acetylcholine in microcirculation) [31].

The group of Schini-Perth published a series of elegant experiments tackling the effects of SGLT2i on the deleterious consequences of ANG2 exposure in experimental settings. In porcine cultured endothelial cells and native endothelial segments, hyperglycemia induced the local angiotensin system and increased the expression of SGLT1 and 2 proteins as well as the markers of oxidative stress and senescence. Since SGLT2 inhibition with empagliflozin affected neither the redox-sensitive NADPH oxidase nor the H_2_O_2_-induced cellular senescence, the authors ruled out its direct antioxidant effect in the vasculature [32]. The same group further investigated the effect of EMPA on hypertension-induced cardiac and vascular dysfunction in a rat model of chronic AII administration via minipumps. They reported that prior administration of EMPA (30 mg/kg in diet) alleviated the AII-induced diastolic dysfunction, abolished the induction of the pro-oxidant pathway AT1 receptor/NADPH oxidases (NOX)/SGLT2 receptors, and improved the endothelial nitric oxid synthase (eNOS)/ROS balance in both micro- and macrovessels. In particular, treatment with EMPA significantly prevented the AII-induced reduction in NOX4 and increase in NOX1, p47phox, p22phox (but not NOX2) expressions [33]. Of note, EMPA has been previously demonstrated to prevent the upregulation of NOX4, the highest abundant NOX isoform, in a murine model of type 2 diabetes [34].

More recently, in a complex and elegant study Kolijn et al. investigated the acute effects of EMPA on myocardial samples from non-diabetic patients with HFpEF and demonstrated the reduction of both oxidative stress and pro-inflammatory pathways. Specifically, they showed that EMPA significantly attenuated H_2_O_2_, 3-nitrotyrosine GSH accumulation, and lipid peroxidation in both cytosol and mitochondria of cardiomyocytes. The drug also prevented eNOS uncoupling (via reduced phosphorylation at Ser1177), and ROS formation with the restoration of soluble guanylate cyclase activity, cyclic guanosine monophosphate concentration, and subsequently the PKGIα activity. The latter effect is responsible for the phosphorylation of several proteins involved in myofilament contraction and stiffness of cardiomyocyte underlying diastolic dysfunction in HFpEF [35]. Of interest, in a classic murine model of HFpEF elicited by high-fat diet and constitutive NOS inhibition with L-NAME, myocardial dysfunction and inflammation were mainly driven by iNOS upregulation [36].

However, other enzymes, such as MAOs, are relevant sources of cardiovascular oxidative stress in cardiometabolic pathologies [37]. We report herein that both MAO-A and MAO-B isoforms were present in the samples harvested from overweight patients. No differences in atrial gene and protein expression of the MAO isoforms were observed. This finding is in line with the results reported in a diet-induced obesity animal model [38] and at variance from experimental diabetes, where an increase in the cardiac MAO-B isoform occurs [39]. We have showed that both EMPA and DAPA could target other enzymatic sources of ROS in the human failing myocardium, such as MAO at the outer mitocondrial membrane, as a class effect occuring in the presence of ANG2 and high GLUC at doses relevant for the clinical scenario. Also, we have demonstrated that the drugs do not act as direct ROS scavengers.

The role of MAO-related oxidative stress in human myocardium has been unequivocally proven in a series of elegant experiments by the group of Ethan Anderson. Thus, they initially reported that the risk of postoperative atrial fibrillation was associated with MAO activity [40]; more recently, they showed that inhibition of cardiac MAO-A elicited an anti-arrhythmic effect by enhancing diastolic Ca^2+^ handling under catecholamine stress [41].

We acknowledge as limitation of the present study the fact that we did not address the signal transduction the beneficial antioxidant effect of the SGLT2i; further studies are mandatory to elucidate the underlying molecular mechanisms. Of interest, in a recent paper Castoldi et al. investigated whether local modulation of the sympathetic nervous system and inflammation mediates the beneficial effects of SGTL2 inhibitors on myocardial fibrosis and hypertrophy in rat model Ang2-dependent hypertension. These authors showed that administration of empagliflozin prevented the upregulation of tyrosine hydroxylase [42], an observation that is worth for further investigation in humans. The observation that MAO is targetable with SGLT2i might be also relevant for the field of oncology, since the catalytic activity of the MAO-A isoform has been associated with the tumorigenesis-driven oxidative stress and several studies have recently showed the anticancer effects of SGLT1 and SGLT2 inhibitors [43].

The group of Coert Zuurbier unequivocally demonstrated that SGLT2i exert beneficial effects on cardiac metabolism and function via the inhibition of the cardiac sodium transporters and the alteration of ion homeostasis, particularly in the setting of heart failure with increased sympathetic stimulation [9]. Specifically, direct inhibition or decreased protein expression of the sodium–hydrogen exchanger 1 (NHE-1) were associated with cardioprotection in various experimental settings; importantly, the infarct size reduction was reported for EMPA in mice knock-out for the SGLT2 expression [44]. The interaction of SGLT2i with MAO in this setting is worth for further investigation.

## Conclusions

In overweight patients, MAO contributes to atrial oxidative stress and can be targeted with empagliflozin and dapagliflozin, as novel off-target class effect of the SGLT2i. Further investigations are required to elucidate the underlying cardioprotective mechanism of these disease-modifying drugs. Repurposing this class of antidiabetics for the treatment of other chronic pathologies is a trend that will increase as much as their mechanisms of action will be unveiled.

## Data Availability

The authors confirm that the data supporting the findings of this study are available within the article.

## References

[1] Marilly E, Cottin J, Cabrera N, Cornu C, Boussageon R, Moulin P, Lega JC, Gueyffier F, Cucherat M, Grenet G (2022) SGLT2 inhibitors in type 2 diabetes: a systematic review and meta-analysis of cardiovascular outcome trials balancing their risks and benefits. Diabetologia 65:2000–2010. 10.1007/s00125-022-05773-8 35925319 10.1007/s00125-022-05773-8

[2] Delgado E, Jódar E, Mezquita-Raya P, Moreno-Pérez Ó (2022) Benefits of SGLT2i for the treatment of heart failure irrespective of diabetes diagnosis: a state-of-the-art review. Diabetes Ther 13:19–34. 10.1007/s13300-022-01278-0 35704165 10.1007/s13300-022-01278-0 PMC9198410

[3] Preda A, Montecucco F, Carbone F, Camici GG, Lüscher TF, Kraler S, Liberale L (2024) SGLT2 inhibitors: from glucose-lowering to cardiovascular benefits. Cardiovasc Res 120:443–460. 10.1093/cvr/cvae047 38456601 10.1093/cvr/cvae047 PMC12001887

[4] McDonagh TA, Metra M, Adamo M, Gardner RS, Baumbach A, Böhm M, Burri H, Butler J, Čelutkienė J, Chioncel O, Cleland JGF, Coats AJS, Crespo-Leiro MG, Farmakis D, Gilard M, Heymans S, Hoes AW, Jaarsma T, Jankowska EA, Lainscak M, Lam CSP, Lyon AR, McMurray JJV, Mebazaa A, Mindham R, Muneretto C, Francesco Piepoli M, Price S, Rosano GMC, Ruschitzka F, Kathrine Skibelund A (2022) 2021 ESC Guidelines for the diagnosis and treatment of acute and chronic heart failure: developed by the task force for the diagnosis and treatment of acute and chronic heart failure of the European society of cardiology (ESC). With the special contribution of the heart failure association (HFA) of the ESC. Eur J Heart Fail 24:4–131. 10.1002/ejhf.2333 35083827 10.1002/ejhf.2333

[5] McDonagh TA, Metra M, Adamo M, Gardner RS, Baumbach A, Böhm M, Burri H, Butler J, Čelutkienė J, Chioncel O, Cleland JGF, Crespo-Leiro MG, Farmakis D, Gilard M, Heymans S, Hoes AW, Jaarsma T, Jankowska EA, Lainscak M, Lam CSP, Lyon AR, McMurray JJV, Mebazaa A, Mindham R, Muneretto C, Francesco Piepoli M, Price S, Rosano GMC, Ruschitzka F, Skibelund AK (2023) 2023 Focused update of the 2021 ESC guidelines for the diagnosis and treatment of acute and chronic heart failure. Eur Heart J 44:3627–3639. 10.1093/eurheartj/ehad195 37622666 10.1093/eurheartj/ehad195

[6] Heidenreich PA, Bozkurt B, Aguilar D, Allen LA, Byun JJ, Colvin MM, Deswal A, Drazner MH, Dunlay SM, Evers LR, Fang JC, Fedson SE, Fonarow GC, Hayek SS, Hernandez AF, Khazanie P, Kittleson MM, Lee CS, Link MS, Milano CA, Nnacheta LC, Sandhu AT, Stevenson LW, Vardeny O, Vest AR, Yancy CW (2022) 2022 AHA/ACC/HFSA guideline for the management of heart failure: a report of the American college of cardiology/American heart association joint committee on clinical practice guidelines. Circulation 145:e895–e1032. 10.1161/cir.0000000000001063 35363499 10.1161/CIR.0000000000001063

[7] Cowie MR, Fisher M (2020) SGLT2 inhibitors: mechanisms of cardiovascular benefit beyond glycaemic control. Nat Rev Cardiol 17:761–772. 10.1038/s41569-020-0406-8 32665641 10.1038/s41569-020-0406-8

[8] Xiang B, Zhao X, Zhou X (2021) Cardiovascular benefits of sodium-glucose cotransporter 2 inhibitors in diabetic and nondiabetic patients. Cardiovasc Diabetol 20:78. 10.1186/s12933-021-01266-x 33827579 10.1186/s12933-021-01266-x PMC8028072

[9] Chen S, Coronel R, Hollmann MW, Weber NC, Zuurbier CJ (2022) Direct cardiac effects of SGLT2 inhibitors. Cardiovasc Diabetol 21:45. 10.1186/s12933-022-01480-1 35303888 10.1186/s12933-022-01480-1 PMC8933888

[10] Lopaschuk GD, Verma S (2020) Mechanisms of cardiovascular benefits of sodium glucose co-transporter 2 (SGLT2) inhibitors: a state-of-the-art review. JACC Basic Transl Sci 5:632–644. 10.1016/j.jacbts.2020.02.004 32613148 10.1016/j.jacbts.2020.02.004 PMC7315190

[11] Myasoedova VA, Bozzi M, Valerio V, Moschetta D, Massaiu I, Rusconi V, Di Napoli D, Ciccarelli M, Parisi V, Agostoni P, Genovese S, Poggio P (2023) Anti-inflammation and anti-oxidation: the key to unlocking the cardiovascular potential of SGLT2 inhibitors and GLP1 receptor agonists. Antioxidants (Basel). 10.3390/antiox13010016 38275636 10.3390/antiox13010016 PMC10812629

[12] Izzo C, Vitillo P, Di Pietro P, Visco V, Strianese A, Virtuoso N, Ciccarelli M, Galasso G, Carrizzo A, Vecchione C (2021) The role of oxidative stress in cardiovascular aging and cardiovascular diseases. Life (Basel). 10.3390/life11010060 33467601 10.3390/life11010060 PMC7829951

[13] Bou-Teen D, Kaludercic N, Weissman D, Turan B, Maack C, Di Lisa F, Ruiz-Meana M (2021) Mitochondrial ROS and mitochondria-targeted antioxidants in the aged heart. Free Radic Biol Med 167:109–124. 10.1016/j.freeradbiomed.2021.02.043 33716106 10.1016/j.freeradbiomed.2021.02.043

[14] Dhalla NS, Elimban V, Bartekova M, Adameova A (2022) Involvement of oxidative stress in the development of subcellular defects and heart disease. Biomedicines 10:393 35203602 10.3390/biomedicines10020393 PMC8962363

[15] Aimo A, Castiglione V, Borrelli C, Saccaro LF, Franzini M, Masi S, Emdin M, Giannoni A (2020) Oxidative stress and inflammation in the evolution of heart failure: from pathophysiology to therapeutic strategies. Eur J Prev Cardiol 27:494–510. 10.1177/2047487319870344 31412712 10.1177/2047487319870344

[16] Sturza A, Muntean DM, Crețu OM (2021) Monoamine oxidase, obesity and related comorbidities: discovering bonds. In: Tappia PS, Ramjiawan B, Dhalla NS (eds) Cellular and biochemical mechanisms of obesity. Springer, Cham, pp 199–213

[17] Lopez-Jimenez F, Almahmeed W, Bays H, Cuevas A, Di Angelantonio E, le Roux CW, Sattar N, Sun MC, Wittert G, Pinto FJ, Wilding JPH (2022) Obesity and cardiovascular disease: mechanistic insights and management strategies. A joint position paper by the World Heart Federation and World Obesity Federation. Eur J Prev Cardiol 29:2218–2237. 10.1093/eurjpc/zwac187 36007112 10.1093/eurjpc/zwac187

[18] Zhou B, Tian R (2018) Mitochondrial dysfunction in pathophysiology of heart failure. J Clin Invest 128:3716–3726. 10.1172/jci120849 30124471 10.1172/JCI120849 PMC6118589

[19] Kaludercic N, Di Lisa F (2020) Mitochondrial ROS formation in the pathogenesis of diabetic cardiomyopathy. Front Cardiovasc Med 7:12. 10.3389/fcvm.2020.00012 32133373 10.3389/fcvm.2020.00012 PMC7040199

[20] Muntean DM, Sturza A, Dănilă MD, Borza C, Duicu OM, Mornoș C (2016) The role of mitochondrial reactive oxygen species in cardiovascular injury and protective strategies. Oxid Med Cell Longev 2016:8254942. 10.1155/2016/8254942 27200148 10.1155/2016/8254942 PMC4856919

[21] Kaludercic N, Arusei RJ, Di Lisa F (2023) Recent advances on the role of monoamine oxidases in cardiac pathophysiology. Basic Res Cardiol 118:41. 10.1007/s00395-023-01012-2 37792081 10.1007/s00395-023-01012-2 PMC10550854

[22] Duicu OM, Lighezan R, Sturza A, Balica R, Vaduva A, Feier H, Gaspar M, Ionac A, Noveanu L, Borza C, Muntean DM, Mornos C (2016) Assessment of mitochondrial dysfunction and monoamine oxidase contribution to oxidative stress in human diabetic hearts. Oxid Med Cell Longev 2016:8470394. 10.1155/2016/8470394 27190576 10.1155/2016/8470394 PMC4846770

[23] Lascu A, Ionică LN, Buriman DG, Merce AP, Deaconu L, Borza C, Crețu OM, Sturza A, Muntean DM, Feier HB (2023) Metformin and empagliflozin modulate monoamine oxidase-related oxidative stress and improve vascular function in human mammary arteries. Mol Cell Biochem 478:1939–1947. 10.1007/s11010-022-04633-8 36583793 10.1007/s11010-022-04633-8

[24] Merce AP, Ionică LN, Bînă AM, Popescu S, Lighezan R, Petrescu L, Borza C, Sturza A, Muntean DM, Creţu OM (2022) Monoamine oxidase is a source of cardiac oxidative stress in obese rats: the beneficial role of metformin. Mol Cell Biochem. 10.1007/s11010-022-04490-5 35723772 10.1007/s11010-022-04490-5

[25] Sturza A, Leisegang MS, Babelova A, Schröder K, Benkhoff S, Loot AE, Fleming I, Schulz R, Muntean DM, Brandes RP (2013) Monoamine oxidases are mediators of endothelial dysfunction in the mouse aorta. Hypertension 62:140–146. 10.1161/hypertensionaha.113.01314 23670301 10.1161/HYPERTENSIONAHA.113.01314

[26] Luna-Marco C, Iannantuoni F, Hermo-Argibay A, Devos D, Salazar JD, Víctor VM, Rovira-Llopis S (2024) Cardiovascular benefits of SGLT2 inhibitors and GLP-1 receptor agonists through effects on mitochondrial function and oxidative stress. Free Radic Biol Med 213:19–35. 10.1016/j.freeradbiomed.2024.01.015 38220031 10.1016/j.freeradbiomed.2024.01.015

[27] Weissman D, Maack C (2021) Redox signaling in heart failure and therapeutic implications. Free Radic Biol Med 171:345–364. 10.1016/j.freeradbiomed.2021.05.013 34019933 10.1016/j.freeradbiomed.2021.05.013

[28] Li A, Zheng N, Ding X (2022) Mitochondrial abnormalities: a hub in metabolic syndrome-related cardiac dysfunction caused by oxidative stress. Heart Fail Rev 27:1387–1394. 10.1007/s10741-021-10109-6 33950478 10.1007/s10741-021-10109-6 PMC9197868

[29] Yaribeygi H, Atkin SL, Butler AE, Sahebkar A (2019) Sodium-glucose cotransporter inhibitors and oxidative stress: an update. J Cell Physiol 234:3231–3237. 10.1002/jcp.26760 30443936 10.1002/jcp.26760

[30] Bodnar P, Mazurkiewicz M, Chwalba T, Romuk E, Ciszek-Chwalba A, Jacheć W, Wojciechowska C (2023) The impact of pharmacotherapy for heart failure on oxidative stress-role of new drugs, flozins. Biomedicines. 10.3390/biomedicines11082236 37626732 10.3390/biomedicines11082236 PMC10452694

[31] Waddingham MT, Sonobe T, Tsuchimochi H, Edgley AJ, Sukumaran V, Chen YC, Hansra SS, Schwenke DO, Umetani K, Aoyama K, Yagi N, Kelly DJ, Gaderi S, Herwig M, Kolijn D, Mügge A, Paulus WJ, Ogo T, Shirai M, Hamdani N, Pearson JT (2019) Diastolic dysfunction is initiated by cardiomyocyte impairment ahead of endothelial dysfunction due to increased oxidative stress and inflammation in an experimental prediabetes model. J Mol Cell Cardiol 137:119–131. 10.1016/j.yjmcc.2019.10.005 31669609 10.1016/j.yjmcc.2019.10.005

[32] Khemais-Benkhiat S, Belcastro E, Idris-Khodja N, Park SH, Amoura L, Abbas M, Auger C, Kessler L, Mayoux E, Toti F, Schini-Kerth VB (2020) Angiotensin II-induced redox-sensitive SGLT1 and 2 expression promotes high glucose-induced endothelial cell senescence. J Cell Mol Med 24:2109–2122. 10.1111/jcmm.14233 30929316 10.1111/jcmm.14233 PMC7011151

[33] Bruckert C, Matsushita K, Mroueh A, Amissi S, Auger C, Houngue U, Remila L, Chaker AB, Park SH, Algara-Suarez P, Belcastro E, Jesel L, Ohlmann P, Morel O, Schini-Kerth VB (2022) Empagliflozin prevents angiotensin II-induced hypertension related micro and macrovascular endothelial cell activation and diastolic dysfunction in rats despite persistent hypertension: role of endothelial SGLT1 and 2. Vascul Pharmacol 146:107095. 10.1016/j.vph.2022.107095 35944842 10.1016/j.vph.2022.107095

[34] Li C, Zhang J, Xue M, Li X, Han F, Liu X, Xu L, Lu Y, Cheng Y, Li T, Yu X, Sun B, Chen L (2019) SGLT2 inhibition with empagliflozin attenuates myocardial oxidative stress and fibrosis in diabetic mice heart. Cardiovasc Diabetol 18:15. 10.1186/s12933-019-0816-2 30710997 10.1186/s12933-019-0816-2 PMC6359811

[35] Kolijn D, Pabel S, Tian Y, Lódi M, Herwig M, Carrizzo A, Zhazykbayeva S, Kovács Á, Fülöp G, Falcão-Pires I, Reusch PH, Linthout SV, Papp Z, van Heerebeek L, Vecchione C, Maier LS, Ciccarelli M, Tschöpe C, Mügge A, Bagi Z, Sossalla S, Hamdani N (2021) Empagliflozin improves endothelial and cardiomyocyte function in human heart failure with preserved ejection fraction via reduced pro-inflammatory-oxidative pathways and protein kinase Gα oxidation. Cardiovasc Res 117:495–507. 10.1093/cvr/cvaa123 32396609 10.1093/cvr/cvaa123

[36] Schiattarella GG, Altamirano F, Tong D, French KM, Villalobos E, Kim SY, Luo X, Jiang N, May HI, Wang ZV, Hill TM, Mammen PPA, Huang J, Lee DI, Hahn VS, Sharma K, Kass DA, Lavandero S, Gillette TG, Hill JA (2019) Nitrosative stress drives heart failure with preserved ejection fraction. Nature 568:351–356. 10.1038/s41586-019-1100-z 30971818 10.1038/s41586-019-1100-z PMC6635957

[37] Sturza A, Muntean DM, Crețu OM (2021) Monoamine oxidase, obesity and related comorbidities: discovering bonds. Cellular and biochemical mechanisms of obesity. Springer, Cham, pp 199–213

[38] Merce AP, Ionică LN, Bînă AM, Popescu S, Lighezan R, Petrescu L, Borza C, Sturza A, Muntean DM, Creţu OM (2023) Monoamine oxidase is a source of cardiac oxidative stress in obese rats: the beneficial role of metformin. Mol Cell Biochem 478:59–67. 10.1007/s11010-022-04490-5 35723772 10.1007/s11010-022-04490-5

[39] Sturza A, Duicu OM, Vaduva A, Dănilă MD, Noveanu L, Varró A, Muntean DM (2015) Monoamine oxidases are novel sources of cardiovascular oxidative stress in experimental diabetes. Can J Physiol Pharmacol 93:555–561. 10.1139/cjpp-2014-0544 25996256 10.1139/cjpp-2014-0544

[40] Anderson EJ, Efird JT, Davies SW, O’Neal WT, Darden TM, Thayne KA, Katunga LA, Kindell LC, Ferguson TB, Anderson CA, Chitwood WR, Koutlas TC, Williams JM, Rodriguez E, Kypson AP (2014) Monoamine oxidase is a major determinant of redox balance in human atrial myocardium and is associated with postoperative atrial fibrillation. J Am Heart Assoc 3:e000713. 10.1161/jaha.113.000713 24572256 10.1161/JAHA.113.000713 PMC3959694

[41] Shi Q, Malik H, Crawford RM, Streeter J, Wang J, Huo R, Shih JC, Chen B, Hall D, Abel ED, Song L-S, Anderson EJ (2024) Cardiac monoamine oxidase-A inhibition protects against catecholamine-induced ventricular arrhythmias via enhanced diastolic calcium control. Cardiovasc Res 120:596–611. 10.1093/cvr/cvae012 38198753 10.1093/cvr/cvae012 PMC11074799

[42] Castoldi G, Carletti R, Ippolito S, Colzani M, Pelucchi S, Zerbini G, Perseghin G, Zatti G, di Gioia CRT (2023) Cardioprotective effects of sodium glucose cotransporter 2 inhibition in angiotensin II-dependent hypertension are mediated by the local reduction of sympathetic activity and inflammation. Int J Mol Sci. 10.3390/ijms241310710 37445888 10.3390/ijms241310710 PMC10341774

[43] Koepsell H (2017) The Na(+)-D-glucose cotransporters SGLT1 and SGLT2 are targets for the treatment of diabetes and cancer. Pharmacol Ther 170:148–165. 10.1016/j.pharmthera.2016.10.017 27773781 10.1016/j.pharmthera.2016.10.017

[44] Chen S, Wang Q, Christodoulou A, Mylonas N, Bakker D, Nederlof R, Hollmann MW, Weber NC, Coronel R, Wakker V, Christoffels VM, Andreadou I, Zuurbier CJ (2023) Sodium glucose cotransporter-2 inhibitor empagliflozin reduces infarct size independently of sodium glucose cotransporter-2. Circulation 147:276–279. 10.1161/circulationaha.122.061688 36649392 10.1161/CIRCULATIONAHA.122.061688

